# Unravelling the Role of Non-Clustered Polyketide Synthases and Key Metabolites in Ochratoxin a Accumulation by *Penicillium nordicum* in Salt-Rich Environments

**DOI:** 10.3390/ijms27177933

**Published:** 2026-09-06

**Authors:** Micaela Álvarez, Jéssica Gil-Serna, Claudio Alba, Elia Roncero, Belén Patiño

**Affiliations:** 1 Sección Departamental de Nutrición y Ciencia de los Alimentos, Facultad de Veterinaria, Universidad Complutense de Madrid, 28040 Madrid, Spain; malvar54@ucm.es (M.Á.); c.alba@ucm.es (C.A.); 2 Departamento de Genética, Fisiología y Microbiología, Facultad de Ciencias Biológicas, Universidad Complutense de Madrid, José Antonio Nováis 12, 28040 Madrid, Spain; belenp@ucm.es; 3 Higiene y Seguridad Alimentaria, Instituto Universitario de Investigación de la Carne y Productos Cárnicos, Facultad de Veterinaria, Universidad de Extremadura, 10003 Cáceres, Spain; eroncerob@unex.es

**Keywords:** gene expression, metabolomics, osmotic stress, mycotoxin biosynthesis

## Abstract

Ochratoxin A (OTA) accumulation by *Penicillium nordicum* is enhanced at high salt concentrations. The genes involved in OTA biosynthesis include a polyketide synthase (PKS), although other PKSs outside the cluster could influence OTA production. This study aimed to evaluate a potential new PKS involved in OTA biosynthesis and the metabolites related to this mycotoxin. Optimised protocols were designed for the gene expression analysis of five genes that encoded the previously reported PKS to be overexpressed in dry-cured meat products (*n2*, *n3*, *n4*, *n5* and *n6*). Their expression was tested in two *P. nordicum* strains (Pn15 and Pn856) grown in CYA medium with different salt concentrations. Fungal mycelia were used for gene expression analyses and metabolomics. Three genes (*n3*, *n4* and *n6*) seem to be related to OTA biosynthesis in Pn856. However, n2 might be involved in OTA degradation. The lack of correlations between the genes studied and OTA in Pn15 could be due to the low OTA levels quantified. The metabolites coniferyl alcohol and 6-hydroxy-8-methoxy-3-methylisochroman were positively correlated to OTA, and 3-methylbutanoic acid and cephalosporolide E might be used as indicators to predict OTA. These results show the complex regulation and influence of PKS proteins on OTA levels.

## 1. Introduction

Ochratoxin A (OTA) is a potent nephrotoxic compound, classified as Group 2B (possible human carcinogen) by the International Agency for Research on Cancer (IARC) [1]. However, new studies suggest its classification in Group 2A (probable human carcinogen) due to its ability to cause kidney tumours in animals and DNA adducts in human cells [1,2].

*Penicillum nordicum* is considered the main OTA producer in dry-cured meat products; its adaptation to NaCl-rich foods seems to be associated with the excretion of this mycotoxin [3]. The molecule of OTA is formed from ochratoxin B through the introduction of a chlorine atom by a halogenase (HAL) enzyme [4]. Therefore, the constant biosynthesis and excretion of OTA, which contains a chloride atom, ensures the maintenance of homeostasis in the fungal cell under high osmolarity conditions [3]. However, other reasons must be behind the synthesis of OTA, since this secondary metabolite is not only excreted in salt-rich environments.

To understand this process, it is essential to analyse its underlying genetic mechanisms. In this sense, Geisen et al. [5] described putative genes involved in OTA synthesis in *P. nordicum* and highlighted *otapksPN*, encoding a polyketide synthase (PKS), and the *npsPN* gene, encoding a non-ribosomal peptide synthetase (NRPS), as key genes. However, these genes do not always correlate with OTA production by *P. nordicum* [6]. In 2011 and 2016, Gil-Serna et al. [7,8] analysed two new genes encoding a PKS and an NRPS in *Aspergillus westerdijkiae* and *Aspergillus steynii*, respectively, and their expression levels also seemed to correlate with OTA production. Finally, these two genes were described to be part of a putative cluster of OTA biosynthesis, not only in *Aspergillus* spp. but also in *P. nordicum.* These genes are clustered together with other ORFs which predict a HAL, a bZIP transcription factor (bZIP), and a cytochrome P450 monooxygenase (P450) [9]. In contrast, it is known that the gene *otapksPN* described by Geisen et al. [5] was located outside the OTA cluster. Therefore, the available information suggests that other genes out of the core cluster can be involved in the OTA synthesis in *P. nordicum* [10]. In this context, previous studies have shown an apparently direct relation between the decrease in the abundance of some PKS enoyl reductase (PKS ER) domain-containing proteins and a reduction in OTA production by *P. nordicum* strains in dry-cured fermented sausage matrices [11,12]. In particular, the use of a biocontrol agent (rosemary essential oil) against *P. nordicum* Pn15 reduced OTA production by up to 80% and decreased the abundance of 21 PKS ER domain-containing proteins [12]. Similarly, the use of another biocontrol agent (*Debaryomyces hansenii* FHSCC 253H), which inhibited OTA production by *P. nordicum* in the dry-cured fermented sausage “salchichón”, also reduced the abundance of nine of these proteins [11]. Furthermore, previous research has related proteins containing this domain to OTA production levels by other moulds such as *Penicillium thymicola* or *Aspergillus niger* [13,14]. Therefore, these studies suggest the potential implication of other PKSs out of the OTA cluster on mycotoxin production, especially in salt-rich meat matrices.

In this context, while previous proteomic insights highlighted the importance of these specific enzymes, an integrated approach combining gene expression and metabolomics provides a more comprehensive understanding regarding the specific changes that occur in the mould. This allows for the evaluation of transcriptional regulation alongside functional metabolic changes. Additionally, the metabolomic profile can contribute to detecting biomarkers, unravelling other metabolic pathways putatively involved in mycotoxin production and revealing the metabolites that are either depleted or overproduced in response to environmental, genetic, pathological, and developmental conditions [15]. Therefore, specific metabolic profiles can detect differences or similarities among different strains of *P. nordicum* and their responses to the same stimuli.

Thus, this work aimed to study the molecular-level relative gene expressions of the genes encoding these new overrepresented PKS ERs to further investigate the relationship between their expressions and OTA production at the NaCl concentrations that usually occur in dry-cured meat products. Additionally, the metabolic profiles of *P. nordicum* strains were assessed in order to detect new metabolites that might be involved in mycotoxin production through untargeted metabolomics and to establish how salt concentration influences the fungal metabolome. By combining gene expression analysis with untargeted metabolomics and incorporating our previous proteomic data, we bridge the gap between transcriptional activation and the final phenotypic metabolic output. This completes the comprehensive analysis, providing an integrated overview of the OTA biosynthetic pathway.

## 2. Results and Discussion

### 2.1. Optimisation of the Real-Time qPCR Protocol for Gene Expression Analysis

The five pairs of primers selected achieved proper amplification efficiencies of between 105 and 111% (Table 1); therefore, all protocols met the criteria (amplification efficiencies close to 100%; a difference in amplification efficiencies of less than 10% between the constitutive and target genes; an amplicon size of cDNA ≤ 150 pb) and, therefore, gene expression was analysed using the 2^−∆∆CT^ method [16,17].

### 2.2. Growth and OTA Production by P. nordicum Strains in Salt-Rich Media

**Figure 1:**
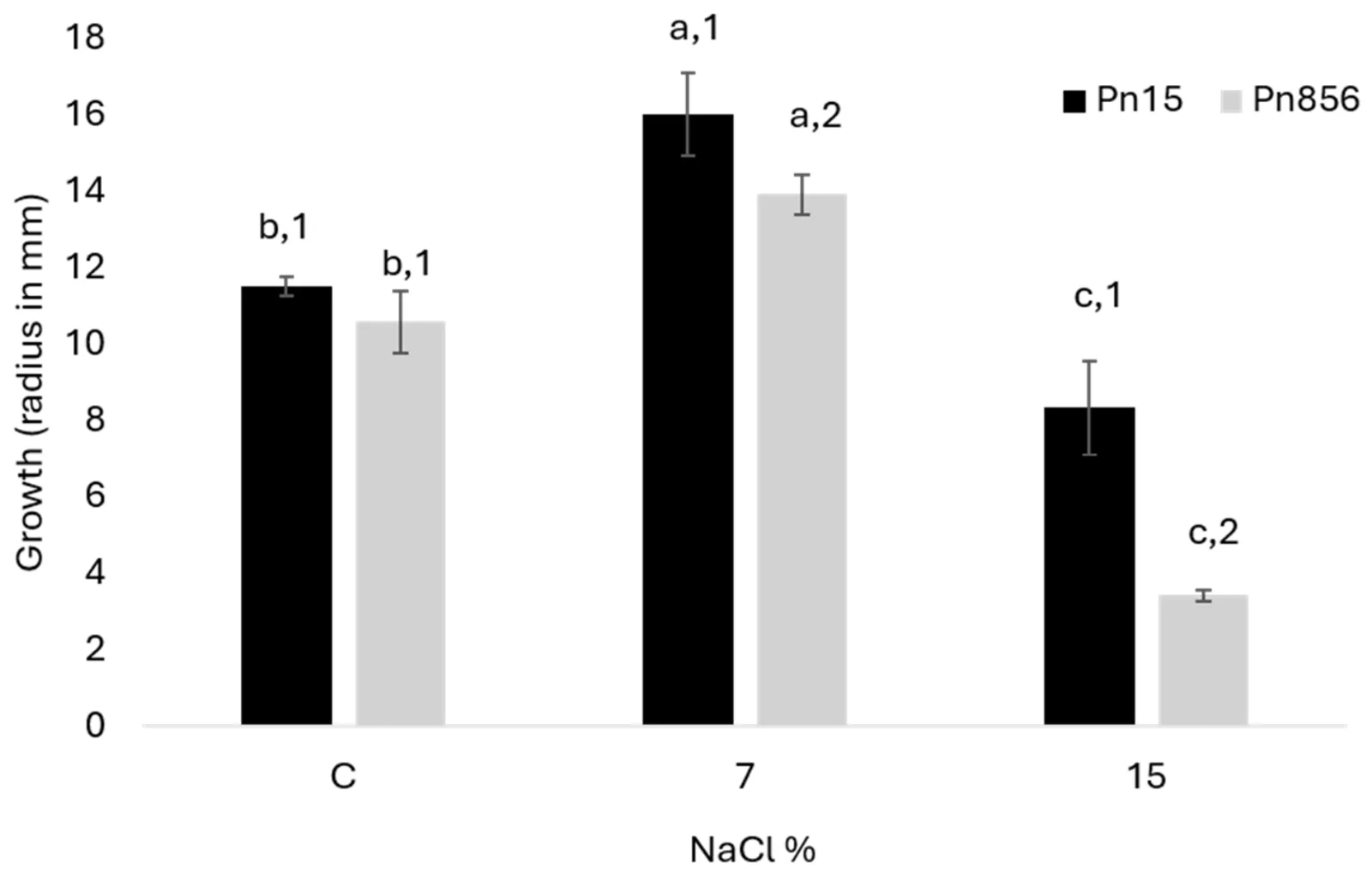
Growth of *Penicillium nordicum* FSHCC Pn15 (Pn15) and BFE 856 (Pn856) after 12 days of incubation in CYA plates supplemented with different NaCl concentrations [control (C), 7% and 15% *w*/*v*]. Different letters indicate statistically significant differences between the control and treatments, while different numbers indicate significant differences between strains within the same treatment (*p* ≤ 0.05).

The results showed that Pn15 formed colonies with significantly larger diameters than those of Pn856 in the presence of NaCl (treatments 7% and 15%; Figure 1). Therefore, a greater adaptation to saline environments could be assumed. This is clearly in accordance with the origins of the strains. Pn15 was isolated from Spanish dry-cured ham which presents this range of salt concentration [18], whereas Pn856 was isolated from Italian dry-cured ham that contains, on average, a salt concentration lower than 5% [19]. Considering this, it is reasonable that the addition of 70 g/L of NaCl (treatment 7%) stimulated the growth of both moulds, but a higher concentration of 150 g/L retarded their growth. These results indicate that, despite the differences between strains, both moulds survive to high salt concentrations. This has been previously tested for several *P. nordicum* strains, including Pn15, which grew in other salt-rich matrices such as a dry-cured-ham-based medium [20,21].

**Figure 2:**
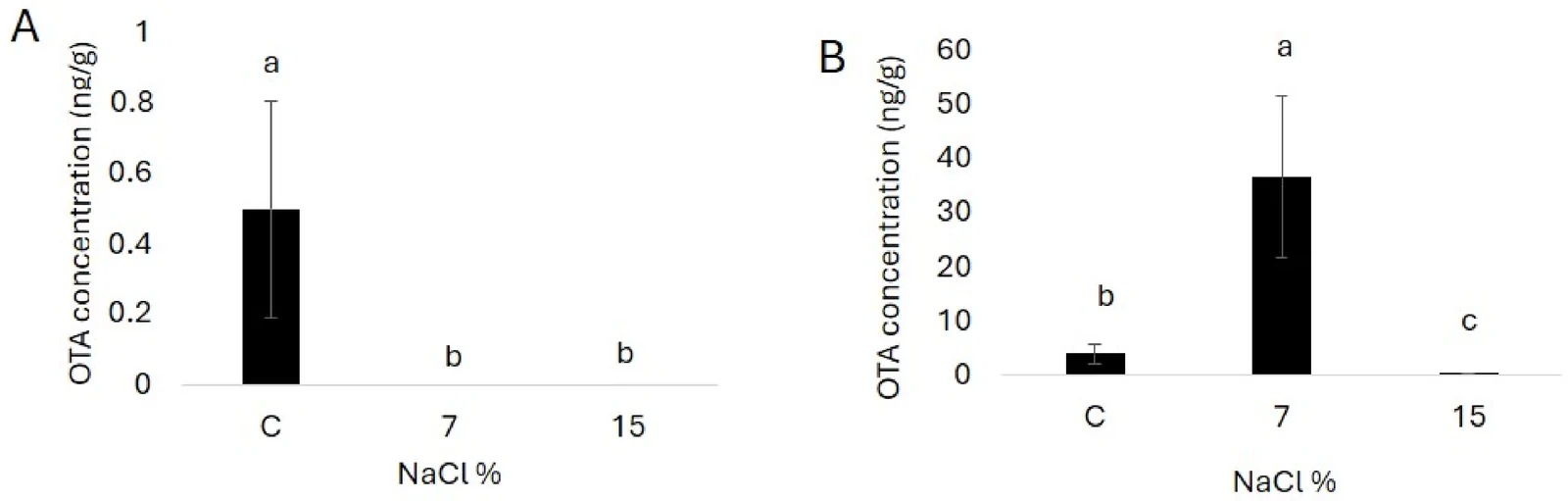
Ochratoxin A concentration produced by *Penicillium nordicum* after 12 days of incubation in CYA medium with different salt concentrations (control, 7% and 15% *w*/*v*). Different letters indicate statistically significant differences between the control (C) and salt treatments (7% and 15%) (*p* ≤ 0.05). (**A**) *P. nordicum* FSHCC (Pn15). (**B**) *P. nordicum* BFE 856 (Pn 856).

Despite being able to grow at all the salt concentrations tested, OTA production ability widely differed between strains and treatments (Figure 2). The Pn856 strain produced higher amounts of the mycotoxin than Pn15, reaching a maximum in the plates supplemented with 70 g/L of NaCl. This could be also related to the origin of the strains. Pn856 could use OTA excretion to deal with stress through the regulation of chloride homeostasis since OTA contains a chloride atom [20]. Following this hypothesis, it should be expected that the highest salt concentration tested would enhance even more OTA production by the moulds. However, this happens in neither Pn856 nor Pn15 with 15% salt. This phenomenon has been previously documented in ochratoxin A producers [6,22]. This might be due to the fact that OTA is not the last metabolite of the pathway. When this mycotoxin reaches a determined concentration, it could be biodegraded by its own producer to generate other compounds as a self-protection mechanism [4]. In this context, it is well known that the OTA-producing species *A. niger* produces the best-characterised enzyme catalysing OTA hydrolysis, ochratoxinase [23], which might open the possibility that other producers could have similar enzymes. Another possibility would be related to the adaptation of the strains to such a high salt concentration. Maybe they are not completely adapted to 15% salt (*w*/*v*) and consequently reduce their growth while concurrently impacting the pathways responsible for the production of secondary metabolites such as OTA. This might be in accordance with previous works which found that some *P. nordicum* strains (Pn15 and Pn69) synthesised lower quantities of OTA in NaCl-supplemented dry-cured-ham-based medium than in the same medium without an extra supplementation of salt [20,24].

### 2.3. Relative Gene Expression Analysis of P. nordicum Strains in Salt-Rich Media

The PKS ER protein sequences were compared to that predicted for the PKS located in the OTA cluster in *P. nordicum*. The alignments revealed a similarity ranging from 55.6% to 61.7% in all five PKS sequences. In a previous study by our research group [9], the percentage of identities among the different genes in the OTA biosynthetic cluster was compared across several *Aspergillus* and *Penicillium* species. Considering that the identity found between the PKS encoding gene of *P. nordicum* and others such as *A. niger* was quite similar (60%), the PKS described in this study might be fully functional for performing the corresponding step in the synthesis of this mycotoxin.

**Figure 3:**
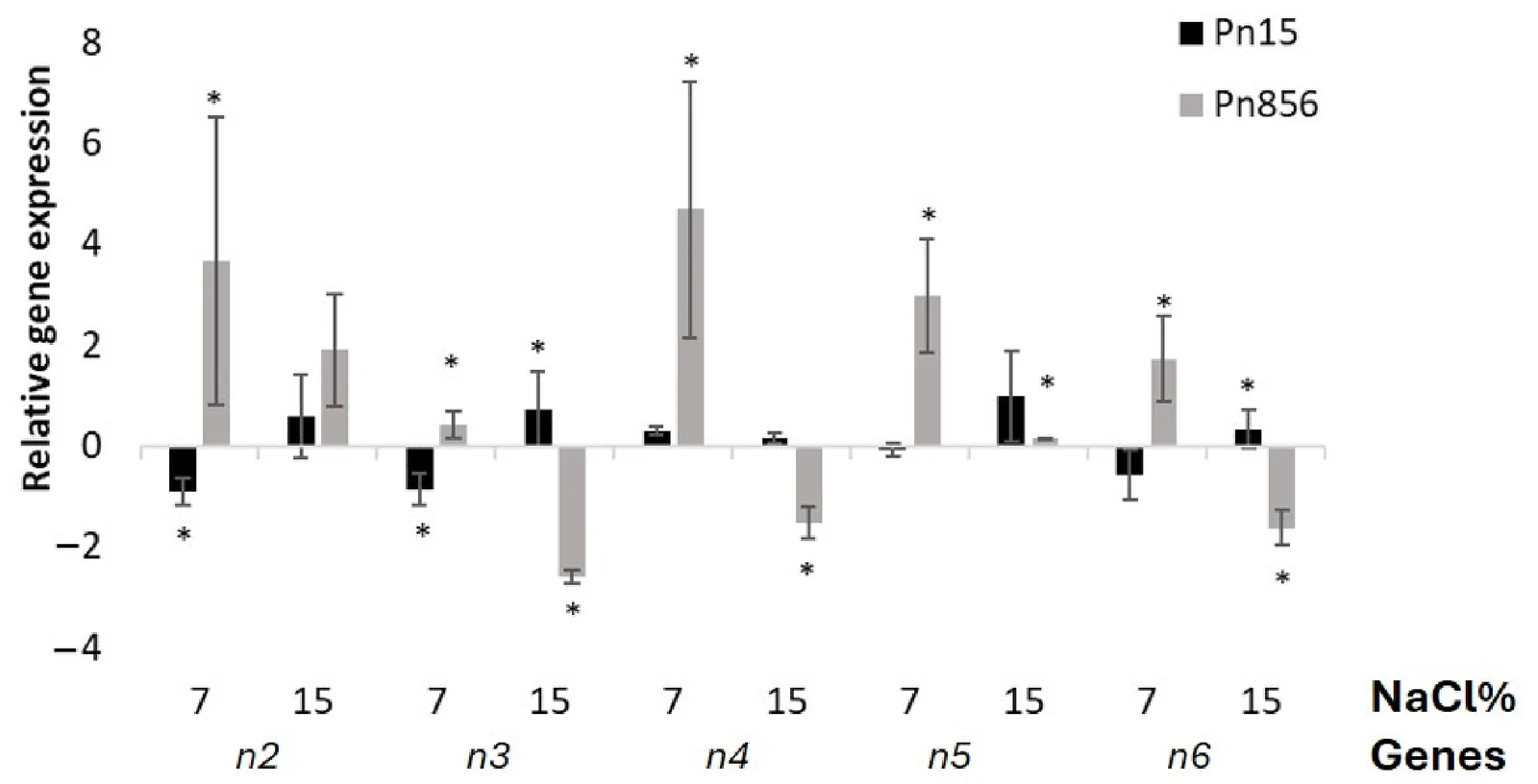
Relative expression of the genes encoding PKS ER-domain-containing proteins (identified by *n2*, *n3*, *n4*, *n5* and *n6*) in *Penicillium nordicum* FSHCC Pn15 (Pn15) and BFE 856 (Pn856) grown for 12 days in CYA medium with different concentrations of NaCl (7% and 15% *w*/*v*). Statistical differences between control batch (calibrator) and treatments are indicated with an asterisk (*p* ≤ 0.05).

The relative gene expressions of the five genes that encode the PKS enzymes with ER domains are shown in Figure 3. In general, there are more changes in the gene expression in Pn856 than in Pn15. The presence of 7% (*w*/*v*) of NaCl caused the overexpression of all the genes studied in the Pn856 strain, whereas the addition of 15% of salt (*w*/*v*) decreased the expression of most of the genes (*n3*, *n4* and *n6*). These results correlated with OTA production since an increase was observed with 7% (*w*/*v*) of NaCl and a decrease with the highest NaCl concentration with respect to the control conditions (Figure 2). The statistical analysis demonstrated strong positive correlations between OTA amounts and the expression of *n3* (ρ = 0.881), *n4* (ρ = 0.915) and *n6* (ρ = 0.865) in Pn856. Therefore, the results might suggest that these three PKS ER proteins might be related to OTA production. Similarly, Wang et al. [25] described a PKS with an ER domain with a similarity of 99% to the *pks* gene of the OTA cluster in *P. nordicum*, and the deletion of this gene completely abolished mycotoxin production. Different authors have suggested that more than one PKS could be needed to synthesize some secondary metabolites, such as zearalenone in *Fusarium graminearum* and the phytotoxic aldaulactone in *Alternaria dauci* [26,27]. On the contrary, no correlation was found between the expression of the genes encoding the new PKS ER and OTA production by Pn15.

The analysis of the upstream and downstream flanks of *n3*, *n4*, *n5* and *n6* revealed the different genes that could be useful in understanding their function (Table 2). The flanking regions of the genes correlated to OTA production in Pn856 (*n3*, *n4* and *n6*) do not reveal the presence of any sequence apparently related to mycotoxin biosynthesis. Interestingly, although the expression of *n2* did not correlate directly with OTA levels, genomic context analyses revealed that *n2* is flanked by genes that encode an amidase and a hydrolase. This result suggests that *n2* might be related to mycotoxin degradation or detoxification rather than its biosynthesis because the amidohydrolases can break the amide bond of the mycotoxin, leading to ochratoxin α and L-β-Phenylalanine [23]. To our knowledge, only amidohydrolases from *A. niger* and some bacteria have been tested for OTA degradation so far [23,28,29]. On the other hand, *n5* function could be involved in the transport of different compounds. Summarising, despite not being directly involved in OTA synthesis, *n2* and *n5* might have an essential role since *n2* could be related to the degradation of OTA amounts or the production of other secondary metabolites derived from polyketides, and *n5* could be involved in their transport across the fungal membrane.

The absence of a clear correlation between the genes *n3*, *n4*, and *n6* and mycotoxin levels in Pn15 may stem from its overall low mycotoxin yield, which potentially mask subtle transcriptional trends. Another possibility could be a rapid transformation or degradation of OTA into other molecules that could hinder the detection of extremely low levels of OTA produced by this strain when NaCl is supplemented to the medium. In this context, Gallo et al. [30] showed that the OTA producer *A. carbonarius* was able to quickly and completely degrade OTA in ochratoxin α and other unknown compounds, with only 14% of OTA remaining after 1 day. Therefore, this lack of correlation could be due to OTA’s fast transformation in other compounds to levels not quantifiable in the 7 and 15% batches (<limit of quantification (LOQ) but >limit of detection (LOD)).

**Figure 4:**
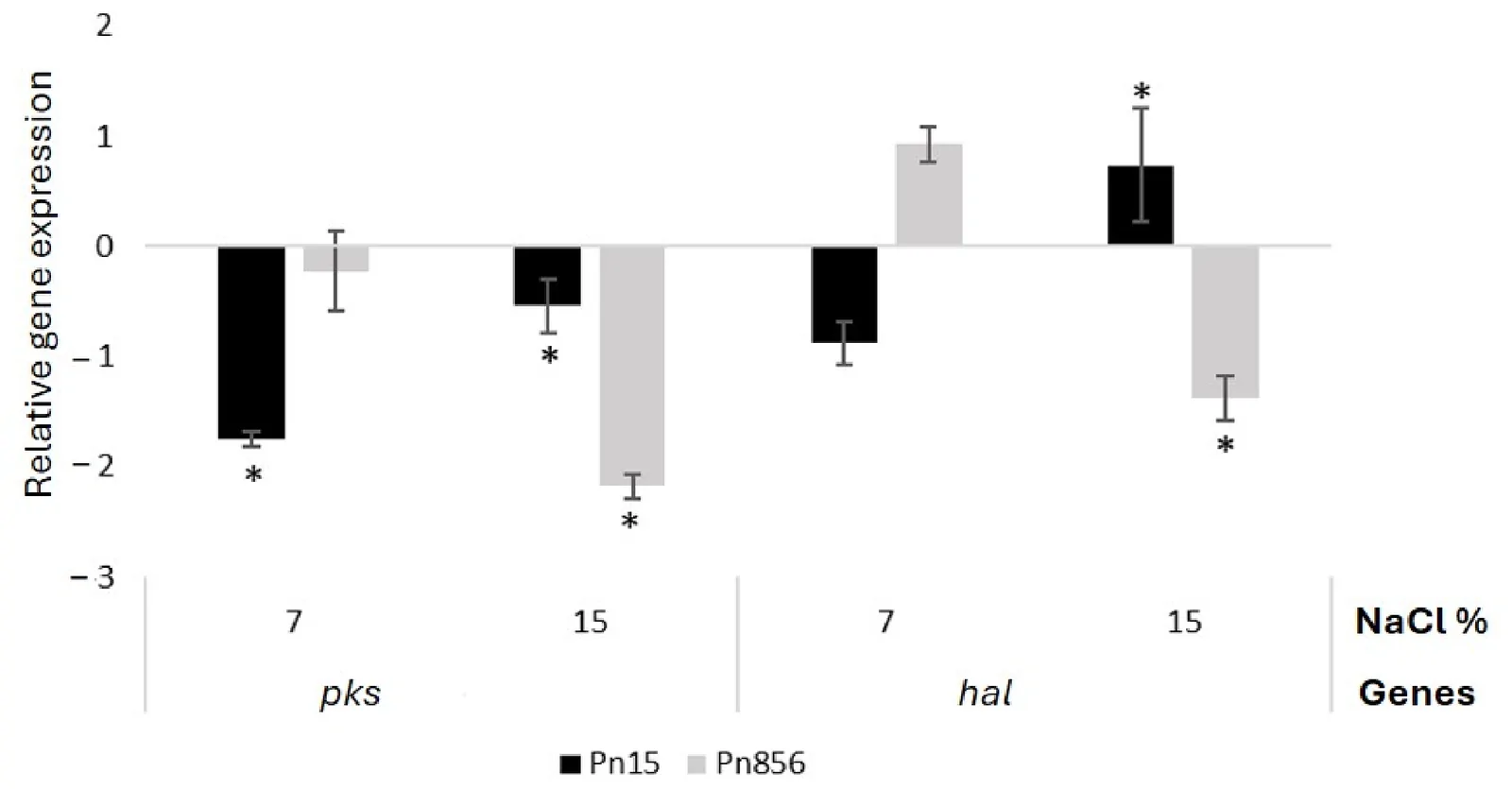
Relative expression of *pks* and *hal* genes in *Penicillium nordicum* FSHCC Pn15 (Pn15) and BFE 856 (Pn856) grown for 12 days in CYA medium with different concentrations of NaCl (7% and 15% *w*/*v*). Statistical differences between the control batch (calibrator) and treatments are indicated with an asterisk (*p* ≤ 0.05).

Regarding the expression of clustered genes involved in the OTA biosynthetic pathway, the gene *pks* was downregulated in most cases with 7 and 15% (Figure 4), showing positive correlations with OTA in both strains (ρ = 0.816 in Pn15 and ρ = 0.729 in Pn856). In fact, the *pks* gene used in this study was selected because it has previously shown positive correlations with OTA production in other *P. nordicum* strains and because it is located in the OTA cluster [9]. Although these correlations were found, the high increase in OTA amounts in the 7% batch of Pn856 does not seem to be associated with an overexpression of this *pks* gene. This result would corroborate the idea that other PKSs can be crucial in OTA biosynthesis. Therefore, the genes that encode other PKSs (*n3*, *n4* and *n6*) which correlated to OTA production might be responsible for this mycotoxin overproduction in Pn856.

In contrast, the gene expression of *hal* was not correlated with the mycotoxin concentrations in Pn15 since it was overexpressed when using 15% (*w*/*v*) of NaCl whereas OTA production was significantly decreased. These results were not expected since the halogenase is involved in the last step of the OTA biosynthetic pathway, introducing a chlorine atom to form the molecule [4]. However, a previous study has demonstrated that this strain (Pn15) deals with osmotic stress by excreting chlorine atoms through the OTA molecules and free chlorine [24]. Therefore, the overexpression of *hal* could be a response to overcome the osmotic stress, excreting the chlorine by alternative routes rather than OTA accumulation. Similarly, in the study described by Wang et al. [31], *A. ochraceus* fc-1 (probably *A. westerdijikiae*) showed the same behaviour as Pn15. Lower OTA amounts produced by the fc-1 strain in a medium with higher NaCl concentrations (7%) than the control batch showed a higher relative gene expression of the halogenase gene *AootaD*.

However, the *hal* expression was indeed correlated to the OTA amounts in Pn856 (ρ = 0.712). Thus, Pn856 could use OTA excretion as the main pathway to overcome the osmotic stress produced by high NaCl concentrations.

### 2.4. Metabolic Profile of P. nordicum Strains in Salt-Rich Media

**Figure 5:**
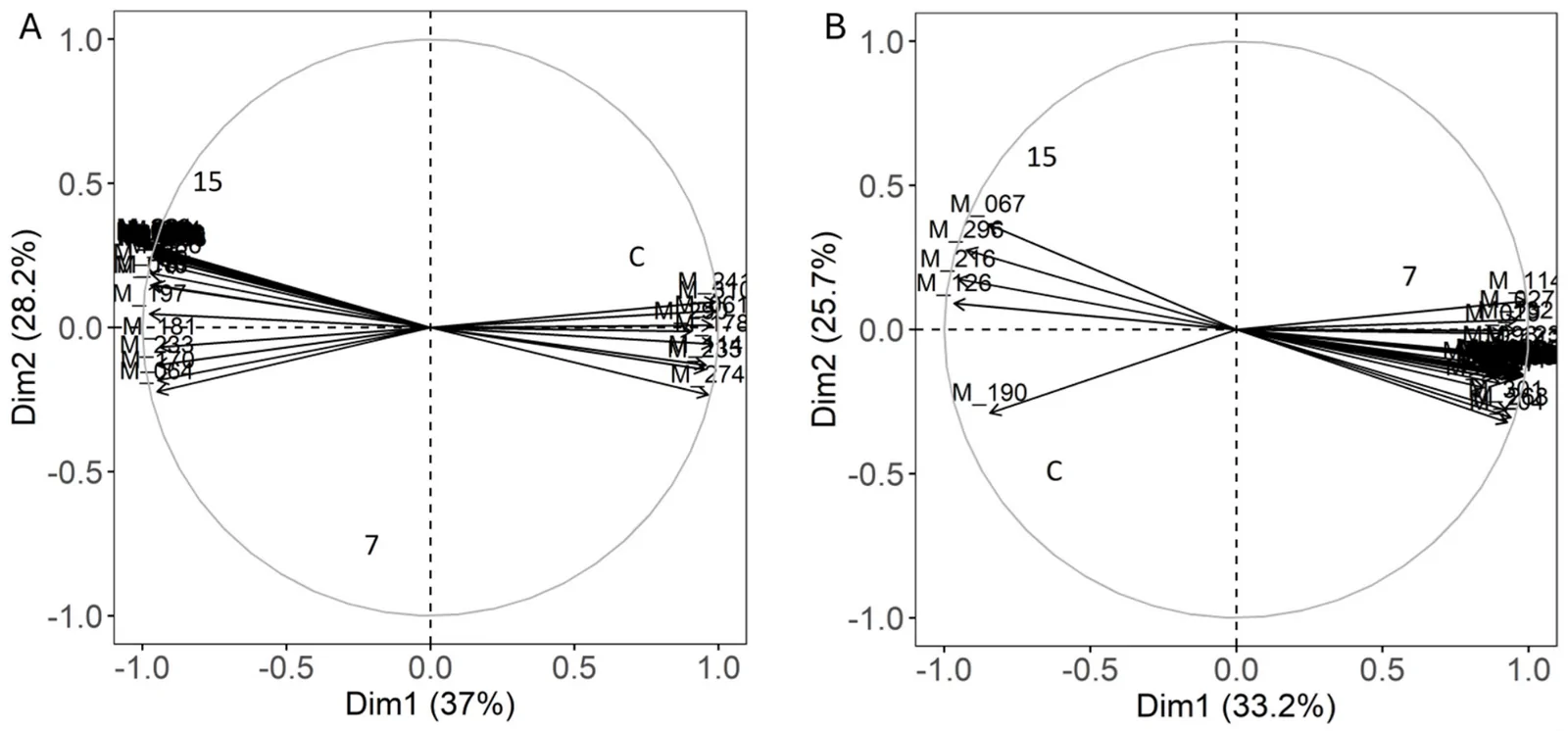
Loading plot after the principal component analysis of metabolites produced by *Penicillium nordicum* strains inoculated in CYA after 12 days. Numbers indicate different compounds as indicated in the Supplementary Materials, Table S1. (**A**) *P. nordicum* FSHCC Pn15. (**B**) *P. nordicum* BFE 856. C: control; 7: batch 70 g/L NaCl; 15: batch 150 g/L NaCl; Dim1: dimension 1; Dim2: dimension 2.

The metabolic profiles of *P. nordicum* under different salt concentrations were determined to identify possible compounds related to its adaptation to NaCl and its ability to produce OTA. This approach allows for a more comprehensive understanding of how salt stress triggers a global secondary metabolic response. The metabolite analyses detected 317 compounds produced by *P. nordicum* strains (Supplementary Materials, Figure S1). The PCA revealed distinct clustering patterns, corresponding to different salt concentrations in each of the two *P. nordicum* strains. The two principal components identified with the results of the Pn15 strain (Figure 5A) accounted for 65% of the total variance in the dataset and for 58.9% in Pn856 (Figure 5B). Therefore, the metabolomic profile revealed that the salt concentrations in the environment strongly affect the production of metabolites by *P. nordicum*, emphasising the differences between strains.

#### 2.4.1. Metabolites of Interest in Pn15

The control batch in Pn15 was characterised by the production of eight compounds including 2,6-Dichloro-4-fluoro-N-methylbenzenesulfonamide, phthalic anhydride, 3-methylbutanoic acid, cephalosporolide E and lyophyllin.

Any metabolite could be used to characterise the 7% batch in Pn15. On the contrary, 41 metabolites characterised the batch supplemented with 15% (*w*/*v*) of NaCl in which OTA was not detected. One of these metabolites is the aspulvinone E. The synthesis of this compound is mediated by two NRPS-like enzymes in *Aspergillus terreus*, and it has been reported to be involved in adaptations to the environment [32]. This suggests that, under the presence of a high salt concentration, Pn15 can produce this compound to better adapt to this stressful stimulus. Another compound detected, botryorhodine B, is known to be cytostatic in moulds such as *A. terreus* and *Fusarium oxysporum* [33], which could be related to the slower mould growth observed in the presence of the highest salt concentration tested.

#### 2.4.2. Metabolites of Interest in Pn856

Regarding the Pn856 strain, only 1 compound had an effect of more than 0.8 in the control batch, with 57 compounds having an effect of more than 0.8 in the 7% batch and 4 in the 15% batch. The high OTA production observed allowed us to clearly differentiate the 7% batch from the rest in this strain. Remarkably, the (3S)-6-hydroxy-8-methoxy-3-methylisochroman was one of the compounds that characterised the batch with 7% of NaCl. This metabolite has been described to be produced by some *Penicillium* spp. adapted to hypersaline environments [34]. Moreover, it showed a positive correlation with the OTA levels (ρ = 0.842; *p* < 0.01). The high amount of (3S)-6-hydroxy-8-methoxy-3-methylisochroman produced by Pn856 in the presence of tolerable NaCl concentrations (7% NaCl) might be related to survival in hyperosmotic conditions, and this adaptation to NaCl could lead to a higher OTA production. This is in accordance with the results of previous authors, who proposed that OTA biosynthesis is an adaptive process for *Penicillium* and *Aspergillus* to counteract high osmolarity conditions due to chloride salts [3,35]. This metabolite was not related to any of the genes studied in Pn856 (*p* > 0.05).

#### 2.4.3. Common Metabolites in Pn15 and Pn856

The other relevant compounds detected were 3-methylbutanoic acid and cephalosporolide E, which characterised the 7% batches in both strains and the control in Pn15. 3-methylbutanoic acid is a volatile compound usually produced by fungi, especially yeasts, and is associated with the flavour of cured food [36,37,38]. A positive correlation of 3-methylbutanoic acid and OTA was found in Pn856 (ρ = 0.733; *p* < 0.05) but not in Pn15. The absence of correlation in Pn15 could be due to the low OTA concentrations quantified in batches 7% and 15%. This suggests that it might be produced under the same stimulus as OTA; thus, future research can explore its function as an OTA biomarker.

As mentioned, cephalosporolide E was also detected in the metabolomic analysis. It is important to highlight that the synthesis of cephalosporolides starts via acetyl-CoA and malonyl-CoA, which overlap with the OTA biosynthetic pathway and are the substrates of the assembly of PKS [39,40]. Therefore, the production of cephalosporolide E might limit mycotoxin production by *P. nordicum*. This result is supported by the strong negative correlation found between both metabolites in Pn15 (ρ = −0.816; *p* < 0.01), since this compound was not detected when OTA was present. However, the correlation between cephalosporolide E and OTA was positive in Pn856, probably due to the high amounts of both compounds found in this strain (ρ = 0.745, *p* < 0.05). These results might indicate that the Pn15 strain might prioritize the synthesis of cephalosporolide E or OTA, considering the lower availability of their precursors (acetyl-CoA and malonyl-CoA). However, Pn856 seems capable of sustaining the simultaneous production of both metabolites at high amounts, depending on whether there are sufficient precursor levels under osmotic stress. The statistical analyses showed no positive correlations between cephalosporolide E and the genes studied in any strain. Thus, the genes that codify the PKS ER studied seem not to be related to the biosynthesis of this metabolite.

Other important compound detected in the metabolomic analysis of both strains is coniferyl alcohol. This molecule has been reported to inhibit fungal growth and, additionally, it is a precursor of coumarin synthesis through the phenylpropanoid biochemical pathway, which is necessary to form the OTA molecule [41]. Interestingly, this compound was only detected in the two batches with the highest OTA production for each strain: the control batch in Pn15 and the 7% batch in Pn856. The results confirmed the positive correlation between this precursor and the OTA molecule in both *P. nordicum* strains (ρ = 0.496; *p* < 0.05). However, the statistical analysis also revealed that the production of this compound was not related to any of the genes that encode the newly described PKS (*n2*, *n3*, *n4*, *n5* and *n6*) (*p* > 0.05).

## 3. Materials and Methods

### 3.1. Fungal Strains and Culture Conditions

Two OTA-producing strains of *P. nordicum* were used in this study: BFE 856 (Pn856), from the Federal Research Centre of Nutrition and Food (Germany), and FHSCC Pn15 (Pn15), from the Food Hygiene and Safety Group at the University of Extremadura (Spain). Pn856 was isolated from Italian Parma ham, whereas Pn15 was obtained from Iberian Spanish ham. Their correct classification was performed by sequencing the ITS1-5.8S-ITS2 region [42]. The isolates were stored in 15% glycerol (Fisher Chemical, UK) at −80 °C until required. These isolates were selected due to their ability to produce OTA in vitro both in laboratory media and in meat-based matrices.

These strains were cultured for 7 days at 25 °C in Potato Dextrose Agar (PDA) to obtain the initial culture. From them, spores were then collected by the addition of 3 mL of sterile saline solution (9 g/L sodium chloride), scraping the plate surface with a sterile spatula. The spores were counted using a Thoma counting chamber Blaubrand^®^ (Brand, Wertheim, Germany), visualising them under a microscope and adjusting to 10^6^ spores/mL.

For the gene expression studies and the metabolomics and OTA detection analyses, 5 µL of each *P. nordicum* spore suspension was cultured in CYA medium (45.4 g/L Czapek-Dox Modified Agar and 5 g/L yeast extract (Pronadisa, Madrid, Spain)) modified with different concentrations of NaCl to simulate the osmotic conditions of dry-cured meat products. Thus, three treatments were tested: C (control without salt), 7% (70 g/L of NaCl) and 15% (150 g/L of NaCl). The radii of the colonies after 12 days of incubation at 25 °C in the three media were measured to compare their fungal growth responses to NaCl. All conditions tested were evaluated in triplicate.

### 3.2. Primer Design and Protocol Optimization

Five PKS ER-domain-containing proteins (Table 1) previously related to OTA production were selected to design the primer [11,12]. The genome sequence of *P. nordicum* DAOMC 185683 was used as reference and obtained from the JGI database. The Uniprot database (https://www.uniprot.org/) was also used to unravel the putative functions of the predicted protein sequences. The sequences of the five PKS ER were compared with the PKS of the OTA biosynthetic gene cluster in *P. nordicum* using LALIGN software (https://www.ebi.ac.uk/jdispatcher/psa/lalign (accessed on 2 March 2026)).

The primer sets were designed based on the 5 sequences of the genes that were used to encode the 5 PKS ER-domain-containing proteins using Primer 3 web version 4.1.0 (https://primer3.ut.ee/ (accessed on 30 November 2025)). All amplified regions contained an intron to allow clear discrimination between the genomic and cDNA templates and the detection of undesired contamination by genomic DNA in RNA samples. The primer length was established between 18 and 23 bp and the melting temperature was set between 62.5 and 66 °C. The absence of harpins and primer dimers was checked using the multiple primer analyser software (Thermo Fisher Scientific, Waltham, MA, USA). In order to maximise amplification efficiency, the amplicon size was established in the range of 100 to 150 bp as recommended in the qPCR optimization guides (https://www.caister.com/highveld/pcr/real-time-pcr-design.html (accessed on 2 March 2026)). The protein identification from Uniprot (ID), ORF1 location, gene name, primer name and sequences, amplicon size and amplification efficiency are described in Table 1.

RNA from the fungal mycelial of both strains was extracted following the methodology described by Gil-Serna et al. [9]. Briefly, the mycelium was scraped from the plate surface with a sterile spatula, frozen with liquid nitrogen and ground using a mortar and a pestle. Cell lysis was carried out using TRI reagent (Ambion, Austin, TX, USA) and the RNA was purified with chloroform and precipitated with LiCl and isopropanol (Sigma-Aldrich, Burlington, MA, USA). RNA concentrations were determined using a NanoDrop^®^ ND-2000c spectrophotometer (Nanodrop Technologies, Wilmington, DE, USA). Retrotranscription was performed using the PrimeScript RT kit starting from 1 µg of total RNA (Takara, Kyoto, Japan).

The analyses of the amplification efficiency, correlation coefficient, and dissociation curve were conducted to confirm the appropriate optimisation of the method. The amplification efficiency of each primer pair at the tested conditions was evaluated by generating a standard curve using the ten-fold serial dilution of cDNA from the Pn856 and Pn15 control plates (batches C). Efficiency was calculated from the slope of the standard curve (%E = (10 − 1/slope − 1) × 100). Real-time qPCR reactions were carried out in an ABI PRISM7900HT system (Applied Biosystems, Madrid, Spain) in the Genomic Unit of the Complutense University of Madrid. The final reaction volume (10 μL) consisted of 5 μL SYBR Premix Ex Taq (Takara, Japan), 0.2 μL ROX, 0.4 μL of each primer 5 μM (Metabion, Boston, MA, USA), 1 μL cDNA template and 3 μL molecular-grade water. The real-time qPCR assays were performed using a standard program: 95 °C for 10 min, 40 cycles at 95 °C for 15 s and 60 °C for 1 min, with a melting curve of 15 s at 95 °C and 15 s at 60 °C, and a 20 min slow ramp between 60 and 95 °C. All reactions were carried out in triplicate.

### 3.3. Quantification of Gene Expression by Real-Time qPCR

Pn856 and Pn15 cultures of 12 days (three replicates) were selected for the gene expression analyses due to their ability to produce high and low OTA concentrations, respectively. cDNA preparation from mycelia growth on CYA plates at the three salt conditions as well as real-time qPCR reactions were carried out as described in Section 2.2. Normalized quantification was performed using the 2^−ΔΔCT^ method [16] and all data were shown to be related to the constitutive β-tubulin gene (*βtub*) expression. In all cases, the results of gene expression are referred to as those obtained in the control batch (C), established as the calibrator to which the value 1 is assigned. Besides the genes that encode the five PKS ER-domain-containing proteins, the halogenase gene (*hal*) and the polyketide synthetase gene (*pks*) expressions were also evaluated as controls since they are genes known to be involved in OTA biosynthesis. The primers used for the gene expression of *βtub*, *hal* and *pks* were described by Gil-Serna et al. [9]. All reactions were evaluated in triplicate.

### 3.4. OTA Quantification and Untargeted Metabolomic Analyses

After 12 days of growth, three agar plugs of 6 mm diameter from both Pn856 and Pn15 in the C, 7% and 15% NaCl batches (*w*/*w*), including the mycelium and agar, were collected for OTA quantification and untargeted metabolomics. The OTA and metabolites extraction employed the QuEChERs procedure previously described by Kamala et al. [43], with modifications by Delgado et al. [20] (mean recovery was around 95%). Briefly, metabolites were extracted with water with acetic acid and acetonitrile acidified with acetic acid 0.1% (*v*/*v*; Fisher Scientific, Waltham, MA, USA) prior to phase partitioning using NaCl (Scharlab S.L, Barcelona, Spain) and anhydrous MgSO_4_. The mixture was vigorously shaken by hand and centrifuged for 5 min at 4 °C at 5300 rpm. Then, the supernatant was analysed by a Q-Exactive Plus Hybrid Quadrupole-Orbitrap Mass Spectrometer (Thermo Fisher Scientific, Waltham, MA, USA) using the method described by Cebrián et al. [44]. Briefly, mobile phases were prepared with HPLC-grade solvents: phase A (H_2_O 0.1% formic acid); phase B (acetonitrile 0.1% formic acid). A measurement of 10 µL was injected and the flow rate was 0.3 mL/min. The ESI source (HESI II, Thermo Fischer Scientific) operated in positive ion mode (ESI+). Data analysis and processing were performed using the FreeStyle™ software, v. 1.5 (Thermo Fisher Scientific). The identification was carried out through its retention time and its exact mass at ≤5 ppm. The LOD and LOQ were 0.0012 ng/mL and 0.01 ng/mL, respectively.

The untargeted metabolomics was performed following the method described by Garrido-Rodríguez et al. [15] using the Q-Exactive Plus equipment described above. The samples stored at −20 °C were diluted at 50% with Milli-Q water to enhance the chromatographic resolution and set in the autosampler to be injected into the HPLC. The identification of the compounds was carried out using Compound Discoverer^®^ software 3.4 [45]. The process involves the generation a mass trace for each detected metabolite, followed by the identification of the compounds through various databases (ChemSpider, mzCloud, Natural Products Atlas 2020_06, and endogenous metabolites database). Identification confidence was assigned following the Metabolomics Standards Initiative (MSI) guidelines. Level 2 (putative identification) was assigned to compounds with a mass error < 5 ppm (measured via Annot. DeltaMass), high FISh Coverage (theoretical fragmentation matching), and consistent matches in the ChemSpider, endogenous metabolites, and Natural Products Atlas databases. For features where experimental MS2 spectra did not yield an exact match in the mzCloud library (mzCloud Best Match Confidence), identification was based on predicted molecular formulas (Predictive Compositions) being classified as Level 3 (putatively characterized classes).

### 3.5. Statistical Analyses

Growth, gene expression analysis, and OTA concentration data were analysed using SPSS software v. 20 (IBM Corporation, Armonk, NY, USA). After testing their normality, data showed a non-normal distribution. Thus, the non-parametric Kruskal–Wallis and Mann–Whitney U tests were applied. The Spearman correlation coefficient was used to evaluate the relations between gene expression and OTA concentration. Statistical significance was established at *p* ≤ 0.05. Principal component analysis (PCA) was conducted to reduce the dimensionality of the normalized metabolite data and to elucidate the primary patterns associated with different salt concentrations and strains. Data were normalized using autoscaling, standardizing each variable to have a mean of zero and a standard deviation of one. The optimal number of clusters was determined using the Gap Statistic method [46]. The PCA, performed using the ‘factoextra’ package in R, included the creation of bi-plots. Only those metabolites contributing a factor greater than 0.8 to the variance in the dataset were displayed in the PCAs, ensuring a focus on the most significant drivers of variation in the metabolomic profiles.

## 4. Conclusions

The results indicate that, despite the differences between strains, both moulds are adapted to high-salt media. Moreover, there might be a new PKS involved in OTA production and its degradation by *P. nordicum* that could help to predict OTA accumulation in salt-rich environments. However, more studies are required to definitively assign their specific roles in OTA production. The metabolic profile demonstrates that different salt levels definitely impact the production of metabolites, highlighting the distinct adaptive strategies between the two strains. Furthermore, some metabolites, such as coniferyl alcohol and the (3S)-6-hydroxy-8-methoxy-3-methylisochroman, might be used as targets of new antifungals to prevent *P. nordicum* contamination and its consequent OTA synthesis in salt-rich matrices such as dry-cured meat products. The results indicate that 3-methylbutanoic acid can be useful to predict OTA levels. Finally, this study demonstrated the need for further research into the broader genomic and regulatory pathways underlying the stress response in *P. nordicum* and its relation to mycotoxin production in order to identify common characteristics across all strains to prevent OTA contamination in dry-cured meat products.

## Figures and Tables

**Table 1:** Primer set designed in this study to be used in real-time -qPCR protocols. Proteins, primer sequences, and amplicon size were generated using DNA and cDNA as templates and the efficiencies of the primers are indicated.

Protein ID	ORF1	Gene Name	Primer Name	Primer Sequence	Amplicon Size DNA/cDNA	Efficiency
A0A0M9WBD1	ACN38_g10787	*n2*	Nord2protF	5′-CAAGCTCCATTCGGTCGGCG-3′	215/140	111%
			Nord2protR	5′-ACTTGGGTTGCGTGCGGTG-3′		
A0A0M8PHV1	ACN38_g1111	*n3*	Nord3protR	5′-CGGCTTCCACGGTACCAGC-3′	206/110	111%
			Nord3protF	5′-GTTGATTAAGGTTGTCGTCGCCG-3′		
A0A0M9WIK6	ACN38_g2830	*n4*	Nord4protF	5′-AGCCACTGGTACTCTTGCCGT-3′	160/111	103%
			Nord4protR	5′-ACCATGTCAGCTCCCCGTGA-3′		
A0A0M9WKW6	ACN38_g296	*n5*	Nord5protF	5′-GACGGGCGAGCAACCTTGAC-3′	192/129	105%
			Nord5protR	5′-TTGGGAAGGAAGCTTGGAGGC-3′		
A0A0N0RYG6	ACN38_g7578	*n6*	Nord6protF	5′-GCCGGCGTCTTTTGCTAGTTG-3′	231/150	106%
			Nord6protR	5′-GAAGAGGCAGCCGGAGTCCC-3′		

**Table 2:** Proteins function that codify the upstream and downstream flanks of the *n2*, *n3*, *n4*, *n5* and *n6* genes.

Gene	Upstream Protein Function	Downstream Protein Function
*n2*	Amidase	Hydrolase
*n3*	Translation	Zinc ion binding
*n4*	-	-
*n5*	ATP binding and transmembrane transport	-
*n6*	-	-

-: No flanked gene detected.

## Data Availability

The data presented in this study are available on request from the corresponding author.

## Supplementary Materials

The following supporting information can be downloaded at: https://www.mdpi.com/article/10.3390/ijms27177933/s1.
